# The Third Annual Meeting of the Southern Surgical and Gynecological Association

**Published:** 1890-12

**Authors:** 


### THE THIRD ANNUAL MEETING OF THE SOUTHERN SURGICAL AND GYNECOLOGICAL ASSOCIATION.

The recent meeting of the Southern Surgical and Gynecological Association, held in this city last month, was by far the most successful in the history of the society. This statement means a great deal, for this Association has by its scientific work won a place second to no body of the kind in this country. It has made itself felt in medical circles all over the United States as is proven by the presence at its recent meeting of men from all sections of the country, who are recognized as leaders in the specialties to which the work of the Association is devoted.

The proceedings throughout were marked by an earnest purpose to advance the sciences of surgery and gynecology, and the amount of work accomplished was a source of surprise and gratification to all who attended the meetings. The programme was fully carried out, and every paper was followed by discussions which were pithy and profitable.

If its future may be judged by its past a brilliant prospect lies before this Association. By following out the policy heretofore observed of rigidly excluding medical politics and adhering strictly to scientific work, it will accomplish an incalculable amount of good, not only for its members and the section in which it is located, but for the profession at large and for the science of medicine.

The next meeting will be held in Richmond, Va. The best wish we can offer the Association is that future meetings may carry on the work which has been so well begun, and that it may continue to hold its high position among the leading medical societies of the land.

